# Effects of endothelial progenitor cells transplantation on hyperlipidemia associated kidney damage in ApoE knockout mouse model

**DOI:** 10.1186/s12944-020-01239-1

**Published:** 2020-03-24

**Authors:** Piyun Gong, Zhongwen Zhang, Dongmei Zhang, Zhiwei Zou, Qian Zhang, Huimei Ma, Jingxiu Li, Lin Liao, Jianjun Dong

**Affiliations:** 1 Department of Endocrinology, Qilu Hospital of Shandong University, Shandong University, Jinan, Shandong 250012 China; 2 Department of Endocrinology and Metabology, the First Affiliated Hospital of Shandong First Medical University, Jinan, 250014 China; 3 Department of Endocrinology and Metabology, Shandong Provincial Qianfoshan Hospital, Cheeloo College of Medicine, Shandong University, Jinan, 250014 China; 4 Department of Cardiovascular Medicine, Ninth Hospital of Xi’an, Xi’an, 710054 China; 5 Quality control office, People’s Hospital of Gaoqing, Zibo, 256300 China

**Keywords:** Hyperlipidemia, Endothelial progenitor cells, Cell transplantation, Kidney damage

## Abstract

**Background:**

Hyperlipidaemia causes kidney damage over the long term. We investigated the effect of the administration of endothelial progenitor cells (EPCs) on the progression of kidney damage in a mouse model of hyperlipidaemia.

**Methods:**

Apolipoprotein E-knockout (*ApoE*^*−/−*^) mice were treated with a high-cholesterol diet after spleen resection. Twenty-four weeks later, the mice were divided into two groups and intravenously injected with PBS or EPCs. Six weeks later, the recruitment of EPCs to the kidney was monitored by immunofluorescence. The lipid, endothelial cell, and collagen contents in the kidney were evaluated by specific immunostaining. The protein expression levels of transforming growth factor-β (TGF-β), Smad2/3, and phospho-Smad3 (p-smad3) were detected by western blot analysis.

**Results:**

*ApoE*^*−/−*^ mice treated with a high-fat diet demonstrated glomerular lipid deposition, enlargement of the glomerular mesangial matrix, endothelial cell enlargement accompanied by vacuolar degeneration and an area of interstitial collagen in the kidney. Six weeks after EPC treatment, only a few EPCs were detected in the kidney tissues of ApoE^*−/−*^ mice, mainly in the kidney interstitial area. No significant differences in TGF-β, p-smad3 or smad2/3 expression were found between the PBS group and the EPC treatment group (TGF-β expression, PBS group: 1.06 ± 0.09, EPC treatment group: 1.09 ± 0.17, *P* = 0.787; p-smad3/smad2/3 expression: PBS group: 1.11 ± 0.41, EPC treatment group: 1.05 ± 0.33, *P* = 0.861).

**Conclusions:**

Our findings demonstrate that hyperlipidaemia causes basement membrane thickening, glomerulosclerosis and the vascular degeneration of endothelial cells. The long-term administration of EPCs substantially has limited effect in the progression of kidney damage in a mouse model of hyperlipidaemia.

## Background

Hyperlipidaemia-associated kidney damage, a progressive disease characterized by fatty degeneration of the kidney, enlargement of the glomerular mesangial matrix and accumulated extracellular matrix, leads to chronic kidney disease and progressive kidney failure [1, 2]. Existing treatments for hyperlipidaemia-associated kidney damage are not satisfactory; for example, medication cannot cure or reverse chronic kidney damage, and maintenance dialysis can result in nausea, low blood pressure, and restless leg syndrome [2]. Thus, the need to find effective approaches to restore kidney function and eventually reduce the progression of kidney damage is critical.

Endothelial cells play a vital role in the maintenance of a normal and healthy vasculature and the endothelial bed [3]. Hyperlipidaemia induces endothelial cell injury accompanied by vacuolar degeneration, and the loss of glomerular endothelium predisposes patients to the activation of platelets, causing slight aneurysmal dilatation of the tubular kidney capillaries, resulting in thickening of the basement membrane, the formation of a dual-track sign, and eventual glomerulosclerosis [1, 2, 4]. Endothelial progenitor cells (EPCs), a type of bone marrow-derived progenitor cell, contribute to endothelial repair and vasculogenesis [5, 6], and the administration of EPCs might be an effective approach for hyperlipidaemia associated with kidney damage. Nevertheless, the potential therapeutic effects of EPCs on hyperlipidaemia-associated kidney damage have not been addressed. A previous study in an acute kidney injury model found that administration of EPCs enhanced microvascular endothelial regeneration and protected against kidney fibrosis [5]; however, Silvestre found that EPC treatment augmented the lesion burden when EPCs were transferred from young to old *ApoE*^*−/−*^ mice [7]. These therapies are still controversial and inconclusive. Thus, we performed this experiment to investigate the effects of EPC treatment on hyperlipidaemia-related kidney disease in an *ApoE*^*−/−*^ mouse model.

## Materials and method

### Animals

Sixteen 6- to 8-week-old male *ApoE*^*−/−*^ C57BL/6 J mice (C57BL/6 J black mice) and eight C57BL/6 J mice were purchased from Vital River (Peking, China). All animal experiments were performed in accordance with the Regulation of Animal Care Management of the Ministry of Public Health, People’s Republic of China (document No. 55, 2001). All animal care and study protocols were approved by the Ethics Committee of Shandong University.

### Splenectomy surgery

To improve the efficiency of EPC transplantation, we followed the protocol by Tousoulis et al. [8] and performed splenectomy before EPC transplantation. The spleens of sixteen *ApoE*^*−/−*^ mice and eight C57BL/6 J mice were excised as previously described [8, 9]. In brief, mice were anaesthetized with an intraperitoneal dose of 0.8% pentobarbital sodium (10 mg/kg body weight). A 10–15 mm incision was made on the left abdomen, and the splenic arteries and venous supply were ligated, after which the spleen was removed. The animal was allowed to recover for 6–7 days before further treatment was performed.

### EPC culture

**Fig. 1:**
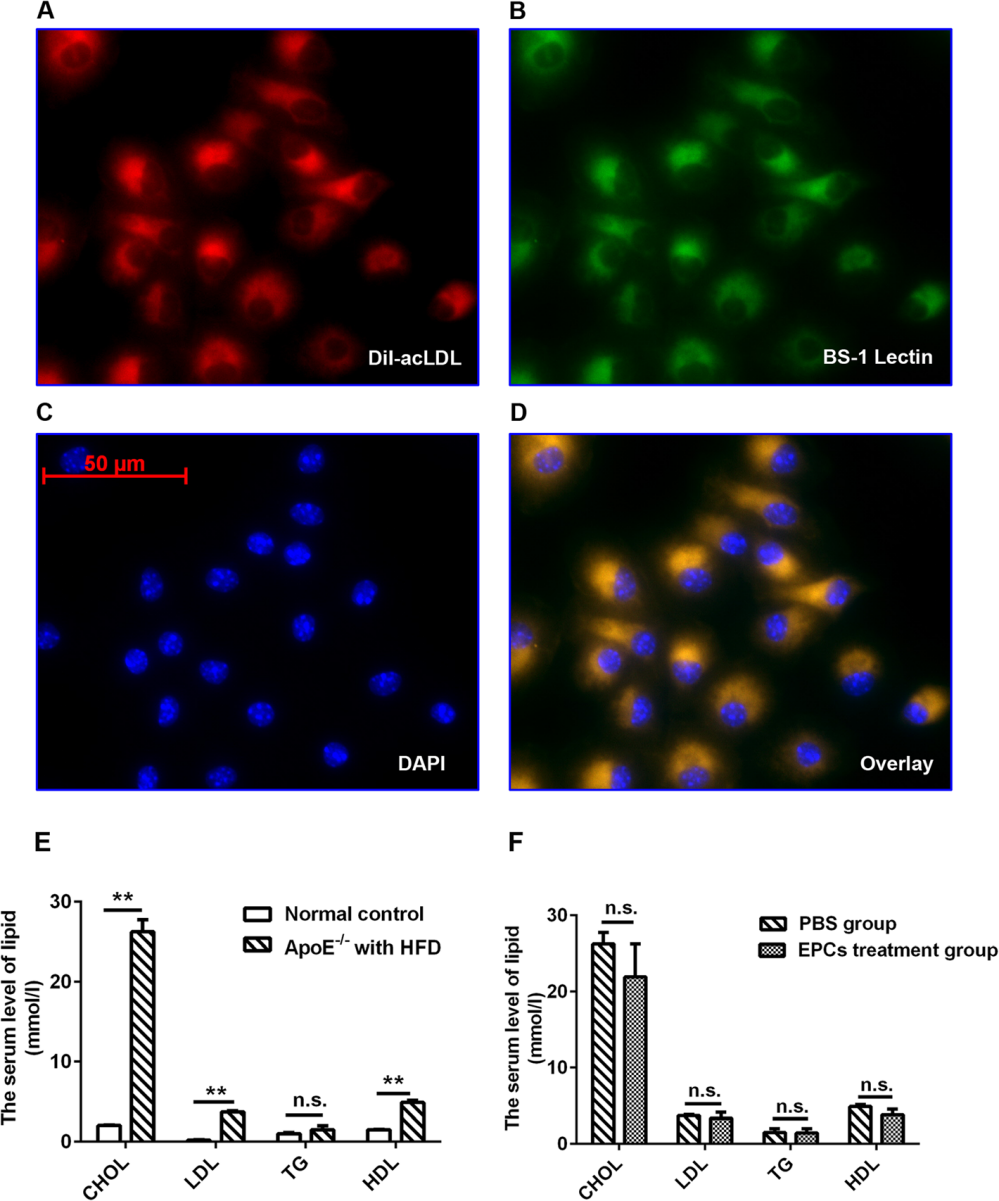
Characterization of EPCs. The serum level of lipid in the normal control mice and ApoE^−/−^ mice after PBS and EPC treatment. Expanded adherent cells could take up (**b**) BS-1 lectin and bind to (**a**) DiI-acLDL as shown by the red and green fluorescence. **c** Nuclei were stained with DAPI (blue). Merged images showed that most cells were (**d**) dual-positive. Dual positive cells were defined as EPCs. The serum level of cholesterol (CHOL), low density lipoprotein (LDL), triglyceride (TG) and high-density lipoprotein (HDL) in the normal control mice, ApoE^−/−^ mice fed on a high-fat diet with PBS or EPC treatment (**e-f**, **P < 0.01; n.s., non-significant; HFD, high fat diet). Bars represent mean ± standard deviation

Bone marrow-derived EPCs were isolated from male C57BL/6 J mice as previously described [10–12]. In short, mononuclear cells were obtained under sterile conditions from the long bones of the mice by flushing with PBS and then purified by the density gradient method (Sigma-Aldrich, St. Louis, MO, USA). Then, the cells were cultured in endothelial cell basal medium-2 (EBM-2) supplemented with reagents from an MV BulletKit (Lonza, Walkersville, MD, USA). Three days later, the medium was replaced with new medium after the nonadherent cells had been removed. After 7 days of culture, EPCs were identified by double fluorescent staining for *Bandeiraea simplicifolia* lectin 1 (BS-1 lectin) and DiI-labelled acetylated low-density lipoprotein (DiI-acLDL) (Sigma-Aldrich) **(**Fig. 1 a-d).

### Preparation of lentivirus vectors and EPC infection

Recombinant lentivirus (Lenti) carrying transgenic EGFP was purchased from GeneChem (Shanghai, China). EPCs at passage 2 were transfected with lentivirus as previously described [12]. Before injection, EPCs carrying EGFP accounted for more than 90% of the total EPCs (data not shown).

### Experimental design

Splenectomized *ApoE*^*−/−*^ mice were treated with a high-fat, high-cholesterol diet (34% sucrose, 21% anhydrous milk fat/butter fat and 0.2% cholesterol) for 24 weeks. Then, the sixteen mice were randomly divided into two groups and intravenously injected with 1 × 10^6^ EPCs carrying EGFP or the same volume of sterile PBS (200 μl). According to the previously reported method [8], the effects of EPCs should be examined in mice that are returned to a normal chow diet after a high-fat/high-cholesterol diet. EPC injection is proposed as a clinical treatment, and this protocol mimics patients’ dietary habits, i.e., once patients start a treatment, their diet should be controlled during the treatment. Therefore, after EPC treatment, animals were returned to a normal chow diet for the remaining experiments. Eight male C57BL/6 J mice treated with a normal chow diet were used as the normal group.

### Immunohistochemical analysis

After 24 weeks of high-fat diet treatment, the twenty-four mice were sacrificed after anesthetization with the intraperitoneal injection of 0.8% pentobarbital sodium as before. The serum levels of cholesterol, triglyceride, low-density lipoprotein and high-density lipoprotein were determined using spectrophotometry. Kidney tissues were collected after saline perfusion and used to prepare paraffin or frozen sections as described previously [8]. Frozen sections were stained with Oil red O stain (Sigma-Aldrich, Santa Clara, CA, USA) to evaluate lipid deposition in the kidney tissues. Paraffin sections were stained with Masson’s trichrome to evaluate areas of collagen fibre proliferation in the kidney tissues. The accumulation of glomerular mesangial matrix was assessed by periodic acid-Schiff (PAS) (Loogene, Beijing, China) staining. The accumulation of EPCs in kidney tissues was evaluated by immunofluorescence analysis as previously described [9]. The peritubular capillary density was determined with anti-CD31 antibody (Abcam, Inc., Cambridge, MA, USA) [9]. The reaction was visualized by staining with 3,3′-diaminobenzidine (DAB) (Abcam, Inc., Cambridge, MA, USA). Images were taken with an Olympus light microscope (Olympus, Tokyo, Japan). Four to five fields for each mouse were visualised in a blinded manner and evaluated using Image-Pro Plus Software (Media Cybernetics, Silver Spring, MD).

### Capillary density

Each renal tissue sample was examined with light microscopy at 400× magnification after staining for CD31 expression (brown). The density of the peritubular capillaries (PTCs) was calculated using a previously published with adaptations [13]. Within the endothelial cell-stained area, we randomly chose ten fields from each slide and viewed them under a 400× microscope. Each field had an area of 0.065 mm^2^. The average number of capillaries/0.065 mm^2^ was used to estimate capillary density [11].

### Western blot analysis

Western blot analysis was performed as previously described [8]. The following primary antibodies were used: rabbit polyclonal anti-β-actin (Neomark, Fremont, CA), rabbit polyclonal anti-Smad2/3 antibody (Cell Signaling Technology, Inc., Boston, USA), rabbit polyclonal anti-phospho-Smad3 (Ser423/425; Cell Signaling Technology, Inc., Boston, USA), and anti-TGF-β antibody (Cell Signaling Technology, Inc., Boston, USA).

### Statistical analysis

Data are expressed as the Mean ± Standard deviation, and statistical analyses were performed with SPSS 25.0 software (SPSS, Inc., Armonk, NY, USA). Comparisons between groups were analysed via unpaired Student’s t-test. Differences for which *P* < 0.05 were considered statistically significant.

## Results

### Contribution of EPCs to angiogenesis in the kidneys of *ApoE*^*−/−*^ mice

**Fig. 2:**
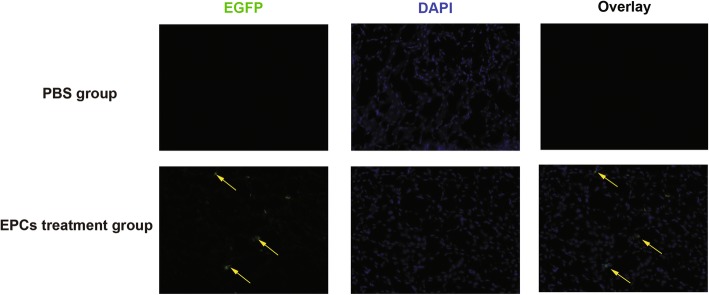
Localization of EPCs in the kidney tissue of ApoE^−/−^ mice. Representative images of EPC incorporation on kidney tissues 6 weeks after PBS and EPC treatment. Nuclei were stained with DAPI (blue). Arrows and arrowheads identify EPCs (green)

To examine the recruitment of EPCs to the kidney tissue, EPCs were transfected with lentivirus encoding EGFP. Before transplantation, isolated cells were identified by double fluorescent staining for BS-1 lectin and DiI-acLDL. As shown in Fig. 1 a-d, isolated cells could take up BS-1 lectin and bind DiI-acLDL, indicating that the isolated cells were EPCs. Six weeks after EPC transplantation, only a few EGFP (+) (labelled putative) EPCs were detected in the kidney tissues of *ApoE*^*−/−*^ mice, mainly in the kidney interstitial area **(**Fig. 2**)**.

**Fig. 3:**
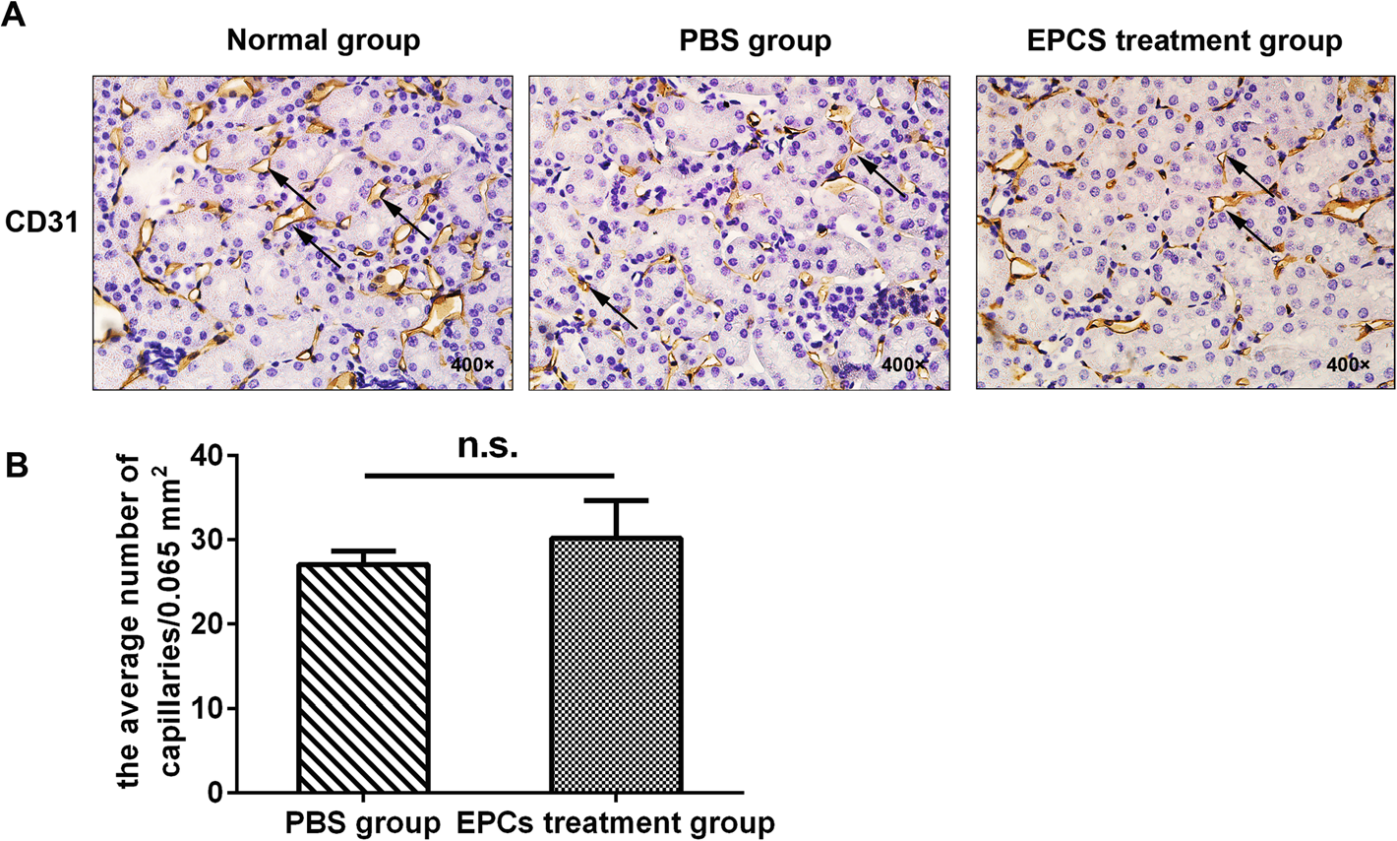
Effect of EPCs transplantation on the kidney capillary density of ApoE^−/−^mice. **a** CD31 staining in the cortical interstitium of the mice 6 weeks after EPCs treatment. **b** Quantitative analysis of capillary density in PBS group and EPCs treatment group (*n* = 4; NS, non-significant). Arrows refer to the endothelium of the luminal side of the cortical interstitium. Bars represent mean ± standard deviation

To investigate whether long-term treatment with EPCs increased the peritubular capillary density, we determined the peritubular capillary density (CD31-positive staining) in *ApoE*^*−/−*^ mice fed a high-fat diet. Fig. 1e shows that the consumption of a high-fat diet for 24 weeks resulted in higher serum cholesterol (*P* < 0.01), low-density lipoprotein (*P* < 0.01) and high-density lipoprotein (*P* < 0.01) levels in the *ApoE*^*−/−*^ mice than in the normal group, indicating that the hyperlipidaemia model had been successfully constructed. There was no evidence that EPCs influenced the serum levels of lipids in the *ApoE*^*−/−*^ group **(**Fig. 1f**)**. As shown in Fig. 3, kidney tubular capillaries exhibited slight aneurysmal dilation, and endothelial cells were enlarged, accompanied by vacuole degeneration, in *ApoE*^*−/−*^ mice fed a high-fat diet. There was an obvious reduction in peritubular capillary density in the *ApoE*^*−/−*^ mice fed a high-fat diet compared to control mice, and the capillary density showed a distinct reduction in tubular atrophy and interstitial expansion. However, disappointingly, when *ApoE*^*−/−*^ mice were treated with EPCs, no significant difference in the peritubular capillary density was detected (PBS group: 27.05 ± 3.23; EPC treatment group: 30.21 ± 8.91, *P* = 0.529).

### Effect of EPCs on dyslipidaemia-induced pathological changes in the kidney

**Fig. 4:**
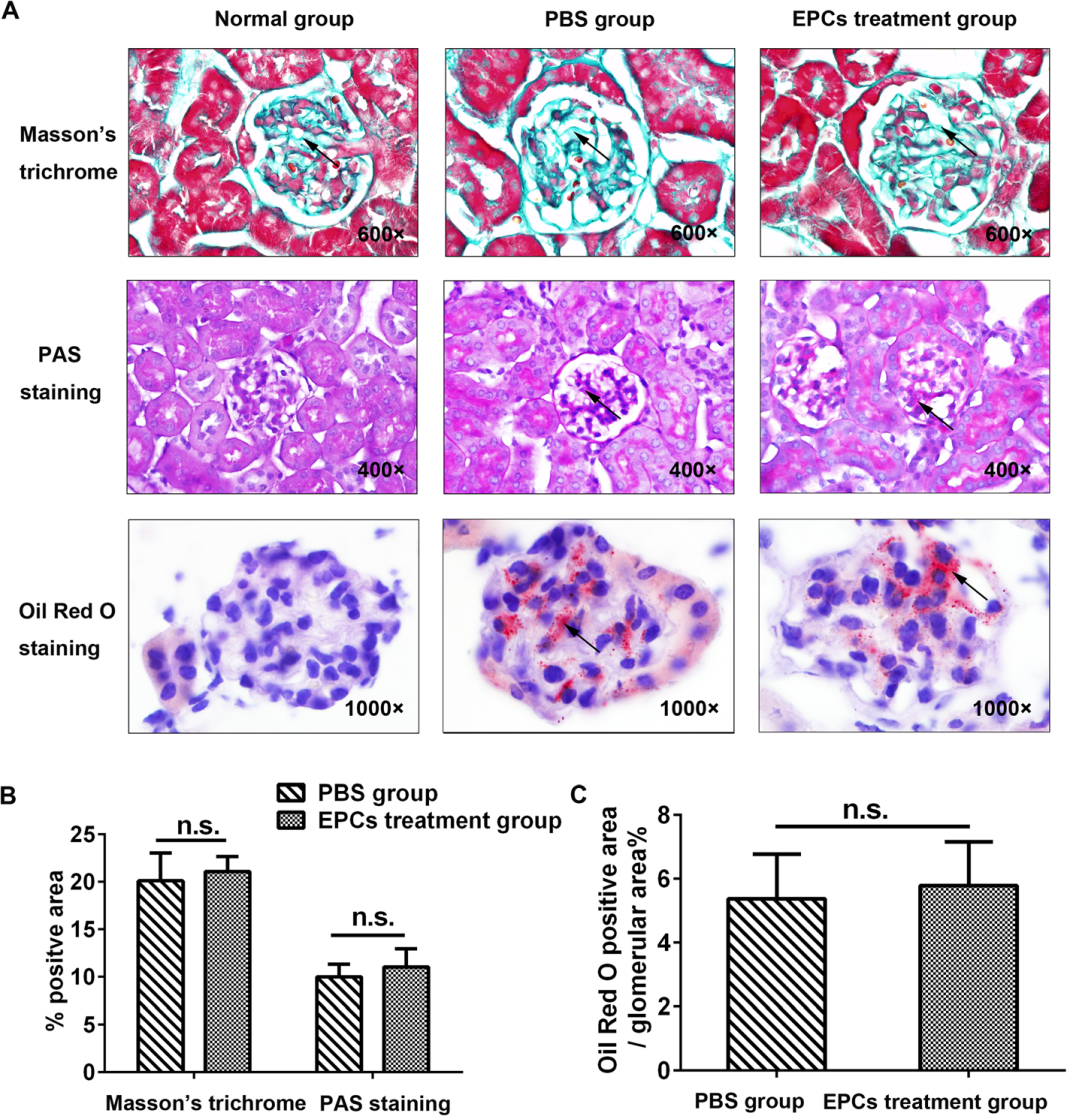
Masson’s trichrome, PAS and Oil Red O staining of kidney tissue from ApoE^−/−^ mice after PBS and EPC treatment. **a** Staining of the collagen, glycogen and lipid in the normal group, PBS group and EPCs treatment group. Arrowheads indicate positive staining areas. **b** Quantitative analysis of **a** (*n* = 4; NS, non-significant). Bars represent mean ± standard deviation

To assess the effects of EPCs on the pathological effects of hyperlipidaemia on the kidney, we examined the lipid burden (the positive area by Oil red O staining) and the accumulation of glomerular mesangial matrix (the positive area by PAS staining) by immunostaining. As shown in Fig. 4, lipid droplets filled the glomerular capillary lumen in the mouse model of hyperlipidaemia, whereas no glomerular staining was observed in mice fed a normal diet. There was no significant difference in positive Oil red O staining between the PBS group and EPC treatment group (PBS group: 5.37 ± 2.42%; EPC treatment group: 5.78 ± 2.38%, *P* = 0.844). Compared to the normal group, *ApoE*^*−/−*^ mice fed a high-fat diet showed an increased mesangial area and glomerular surface area and thickening of the Bowman’s capsule and basement membranes, as evidenced by the increased positively stained area determined by PAS staining and Masson’s trichrome staining. However, there were no statistically significant differences in these parameters between the PBS and EPC treatment groups (PBS group: 9.99 ± 2.33%; EPC treatment group: 11.02 ± 3.34%, *P* = 0.683).

### Effect of EPCs on kidney remodelling in the mouse model of hyperlipidaemia

**Fig. 5:**
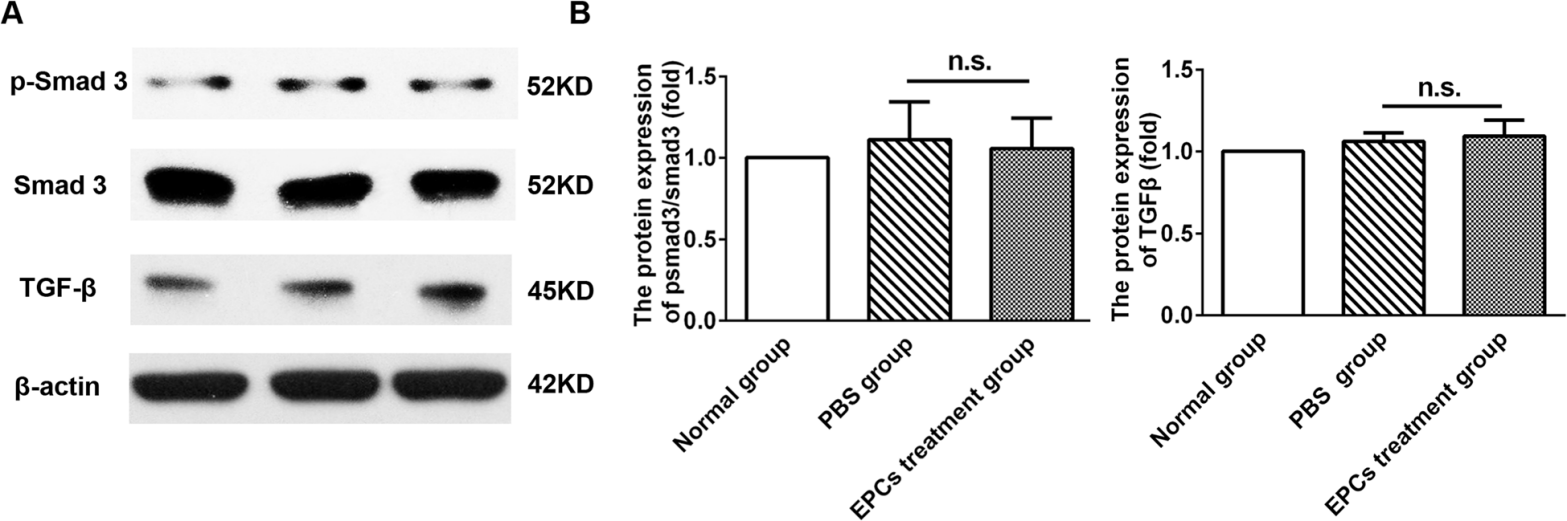
The effects of EPCs treatment on the protein expressions of smad 2/3 and TGF-β in the kidney tissue of ApoE^−/−^ mice (**a)** Relative p-smad 3, smad 2/3 and TGF-β protein expressions measured by western blot in 3 groups of mice. **b** Quantitative analysis of **a** (*n* = 3; NS, non-significant). Data were standardized to β-actin levels after being quantified by phosphor imaging. Representative blots were shown in the upper panel. Bars represent mean ± standard deviation

Most forms of progressive kidney disease eventually develop into kidney fibrosis and remodelling. TGF-β and p-smad3 are the most commonly used markers of kidney differentiation, development and interstitial fibrosis. Thus, we examined the protein expression levels of TGF-β, p-smad3 and smad 2/3 in the mouse kidneys. Additionally, Masson’s trichrome staining was used to evaluate the collagen content of the mouse kidneys. Compared to the normal group, *ApoE*^*−/−*^ mice treated with a high-fat diet exhibited an increased collagen content in the glomeruli, tubules and interstitium **(**Fig. 4a**)**. However, no significant difference in collagen content or TGF-β, p-smad3 or smad2/3 expression was found between the PBS group and EPC treatment group (Masson’s trichrome, PBS group: 20.11 ± 5.84%, EPC treatment group: 21.07 ± 3.16%, *P* = 0.782; TGF-β expression, PBS group: 1.06 ± 0.09, EPC treatment group: 1.09 ± 0.17, *P* = 0.787; p-smad3/smad2/3 expression: PBS group: 1.11 ± 0.41, EPC treatment group: 1.05 ± 0.33, *P* = 0.861, Fig. 4b and Fig. 5a-b).

## Discussion

Although EPC treatment has been heralded as a promising therapeutic strategy for patients with peripheral vascular disease [10], the effect of EPC treatment has not been studied previously in an animal model of hyperlipidaemia. Previous animal studies focused on unilateral ureteral obstruction-induced kidney fibrosis and acute kidney ischaemia rather than hyperlipidaemia-associated kidney damage [11, 14–16]. Nevertheless, hyperlipidaemia-associated kidney damage is present in most clinical situations in which the mobilization of EPCs is planned. The current study is the first to evaluate the effect of the long-term administration of EPCs on the progression of kidney damage in a mouse model of hyperlipidaemia. In this study, we found that the administration of EPCs failed to exert an obvious therapeutic effect on the progression of kidney damage. These data raise the possibility that EPCs are ineffective in the treatment of hyperlipidaemia-associated kidney damage.

*ApoE*, a constituent of very low-density lipoprotein synthesized in the liver and other tissues, serves as a high-affinity ligand for ApoE receptors and is thus responsible for the cellular uptake of lipoprotein particles [17]. Tomiyama-Hanayama et al. showed that eight-week-old *ApoE*^*−/−*^ mice fed a high-fat diet for four weeks exhibited striking lipid deposition and foam cell formation in their glomeruli [18]. *ApoE*^*−/−*^ mice at 36 weeks of age showed more severe renal injury than 24-week-old mice [19]. Consistent with previous studies, we found that in 6- to 8-week-old *ApoE*^*−/−*^ mice fed a high-fat diet for 24 weeks, the mesangial area and glomerular surface area were increased, kidney tubular capillaries exhibited slight aneurysmal dilation, and endothelial cells were enlarged, accompanied by vacuole degeneration.

Hyperlipidaemia leads to clogged arteries, which can ultimately cause cardio-cerebrovascular diseases [20–22]. Further studies have shown that hyperlipidaemia can also cause direct injury to the kidneys, leading to lipid kidney toxicity [23–25]. Therefore, despite the relative paucity of data on the therapeutic effects of EPCs on hyperlipidaemia-associated kidney damage, prior atherosclerosis studies provide ample support for the use of EPCs in animal models of hyperlipidaemia. In a mouse model of hypertension-hypercholesterolemia, the administration of EPCs reduced hepatic lipid accumulation and consequently alleviated hypertension and dyslipidaemia [26]. In contrast, Silvestre et al. found that EPC treatment aggravated the atherosclerosis burden when EPCs were transferred from young to old *ApoE*^*−/−*^ mice [7]. Therefore, the results of prior atherosclerosis studies conflict in terms of both the potential benefits and adverse outcomes of EPC treatment. *Circulation* [27] noted that this controversy is mainly attributed to the very limited recruitment of stem cells to the lesion site. Although stem cells were injected into the injured site, stem cells that gradually aged with increasing treatment time did not exhibit therapeutic efficacy. This is why although the clinical use of stem cell therapies is effective over the short term, outcomes indicate that stem cell therapies are not effective over the long term.

Prior animal studies of the effects of EPCs on kidney-associated diseases are somewhat limited, with one prior study showing that the transplantation of EPCs in a murine model of unilateral ureteral obstruction had a protective effect [11]. Similarly, the long-term administration of EPCs was found to increase the kidney microvasculature, protecting the stenotic kidney in experimental renovascular disease [28, 29]. Nevertheless, these were acute injury models that may not recapitulate the type of effects of hyperlipidaemia-mediated kidney damage.

Most forms of progressive kidney disease eventually develop into kidney fibrosis, and TGF-β and p-smad3, the most important fibrotic factors, can increase degradation of the extracellular matrix (ECM), inhibit ECM synthesis and contribute to cellular epithelial to mesenchymal trans differentiation [30–32]. In this study, we found that administration of EPCs had no influence on the expression of TGF-β and p-smad3 in the kidney tissues of *ApoE*^*−/−*^ mice. The exact mechanism by which EPCs ameliorate kidney fibrosis has not been fully elucidated. As reported by Shuai L et al., kidney fibrosis was improved through the transplantation of bone marrow-derived EPCs, which increased the capillary density and restored angiogenic activity [33]. In this study, we found that very few transplanted EPCs gathered in the kidney lesions of hyperlipidaemic mice and that their role in promoting angiogenesis was very limited. Therefore, the expression levels of TGF-β and p-smad3 were not significantly different in *ApoE*^*−/−*^ mice treated with EPCs compared to control mice.

Our study has several limitations. First, we only studied a single therapeutic dose of EPCs on hyperlipidemia associated kidney damage, additional experiments are required to explore the effect of different therapeutic dose of EPCs on hyperlipidemia associated kidney damage. Second, the process of EPCs homing and mobilizing to the injured site is very complex, further studies are needed to illuminate the inadequate kidney repair achieved by EPCs in ApoE−/− mice.

## Conclusions

Our findings demonstrate that hyperlipidaemia causes basement membrane thickening, glomerulosclerosis and the vascular degeneration of endothelial cells. The long-term administration of EPCs substantially had a limited effect in the progression of kidney damage in a mouse model of hyperlipidaemia. We assessed a new method to improve renal fibrosis, and although its effectiveness was limited, our data have implications for other researchers. Additionally, we are carrying out further studies to validate our findings and delineate their causes.

## Data Availability

The datasets generated during and/or analyses during the current study are available from the corresponding author on reasonable request.

## Abbreviations

EPCs: Endothelial progenitor cells
*ApoE*: Apolipoprotein E knockout
TGF-β: Transforming growth factor-β
p-smad3: Phospho-Smad3
EBM-2: Endothelial cell basal medium-2
BS-1 lectin 1: Bandeiraea simplicifolia lectin 1
DiI-acLDL: DiI-labeled acetylated low-density lipoprotein
Lenti: Lentiviruses
PAS: Periodic acid-schiff
PTCs: Peritubular capillaries
ECM: Extracellular matrix

## References

[1] Davignon J (2005). Apolipoprotein E and atherosclerosis: beyond lipid effect. Arterioscler Thromb Vasc Biol.

[2] Cases A, Coll E. Dyslipidemia and the progression of renal disease in chronic renal failure patients. Kidney Int Suppl. 2005:S87–93. 10.1111/j.1523-1755.2005.09916.x. 10.1111/j.1523-1755.2005.09916.x 16336584

[3] Kang DH, Kanellis J, Hugo C, Truong L, Anderson S, Kerjaschki D (2002). Role of the microvascular endothelium in progressive renal disease. J Am Soc Nephrol.

[4] Hickson LJ, Eirin A, Lerman LO (2016). Challenges and opportunities for stem cell therapy in patients with chronic kidney disease. Kidney Int.

[5] Ishida Y, Kimura A, Kuninaka Y, Inui M, Matsushima K, Mukaida N (2012). Pivotal role of the CCL5/CCR5 interaction for recruitment of endothelial progenitor cells in mouse wound healing. J Clin Invest.

[6] Wang R, Zhang K, Li S, Tong Z, Li G, Zhao Z (2013). Apolipoprotein (a) impairs endothelial progenitor cell-mediated angiogenesis. DNA Cell Biol.

[7] Kundu N, Domingues CC, Chou C, Ahmadi N, Houston S, Jerry DJ, Sen S. Use of p53-silenced endothelial progenitor cells to treat ischemia in diabetic peripheral vascular disease. J Am Heart Assoc. 2017;6. 10.1161/JAHA.116.005146. 10.1161/JAHA.116.005146 PMC5533015 28365567

[8] Tousoulis D, Briasoulis A, Vogiatzi G, Valatsou A, Kourkouti P, Pantopoulou A (2013). Effects of direct infusion of bone marrow-derived progenitor cells and indirect mobilization of hematopoietic progenitor cells on atherosclerotic plaque and inflammatory process in atherosclerosis. Int J Cardiol.

[9] Lima LC, Porto ML, Campagnaro BP, Tonini CL, Nogueira BV, Pereira TM (2012). Mononuclear cell therapy reverts cuff-induced thrombosis in apolipoprotein E-deficient mice. Lipids Health Dis.

[10] Asahara T, Murohara T, Sullivan A, Silver M, van der Zee R, Li T (1997). Isolation of putative progenitor endothelial cells for angiogenesis. Science (New York, NY).

[11] Ma YY, Sun D, Li J, Yin ZC (2010). Transplantation of endothelial progenitor cells alleviates renal interstitial fibrosis in a mouse model of unilateral ureteral obstruction. Life Sci.

[12] Zhang Z, Dong J, Lobe CG, Gong P, Liu J, Liao L (2015). CCR5 facilitates endothelial progenitor cell recruitment and promotes the stabilization of atherosclerotic plaques in ApoE^*−/−*^ mice. Stem Cell Res Ther.

[13] Ohashi R, Kitamura H, Yamanaka N (2000). Peritubular capillary injury during the progression of experimental glomerulonephritis in rats. J Am Soc Nephrol.

[14] Xue J, Qin Z, Li X, Cao P, Jia R (2017). Protective effects of ischemic preconditioning-mediated homing of endothelial progenitor cells on renal acute ischemia and reperfusion injury in male rats. Ann Transplant.

[15] Liang CJ, Shen WC, Chang FB, Wu VC, Wang SH, Young GH (2015). Endothelial progenitor cells derived from wharton's jelly of human umbilical cord attenuate ischemic acute kidney injury by increasing vascularization and decreasing apoptosis, inflammation, and fibrosis. Cell Transplant.

[16] Patschan D, Schwarze K, Tampe B, Zeisberg M, Patschan S, Muller GA (2017). Endothelial Colony forming cells (ECFCs) in murine AKI - implications for future cell-based therapies. BMC Nephrol.

[17] Jawień J, Nastałek P, Korbut R (2004). Mouse models of experimental atherosclerosis. J Physiol Pharmacol.

[18] Tomiyama-Hanayama M, Rakugi H, Kohara M, Mima T, Adachi Y, Ohishi M, Katsuya T, Hoshida Y, Aozasa K, Ogihara T, Nishimoto N (2009). Effect of interleukin-6 receptor blockage on renal injury in apolipoprotein E-deficient mice. Am J Physiol Renal Physiol.

[19] Wen M, Segerer S, Dantas M, Brown PA, Hudkins KL, Goodpaster T (2002). Renal injury in apolipoprotein E-deficient mice. Lab Invest.

[20] Munshi RP, Joshi SG, Rane BN (2014). Development of an experimental diet model in rats to study hyperlipidemia and insulin resistance, markers for coronary heart disease. Indian J Pharmacol.

[21] Eirin A, Zhu XY, Ebrahimi B, Krier JD, Riester SM, van Wijnen AJ (2015). Intrarenal delivery of Mesenchymal stem cells and endothelial progenitor cells attenuates hypertensive cardiomyopathy in experimental Renovascular hypertension. Cell Transplant.

[22] Herz J, Sabellek P, Lane TE, Gunzer M, Hermann DM, Doeppner TR (2015). Role of neutrophils in exacerbation of brain injury after focal cerebral ischemia in hyper-lipidemic mice. Stroke.

[23] Gervais M, Pons S, Nicoletti A, Cosson C, Giudicelli JF, Richer C (2003). Fluvastatin prevents renal dysfunction and vascular NO deficit in apolipoprotein E-deficient mice. Arterioscler Thromb Vasc Biol.

[24] Zhang X, Urbieta-Caceres VH, Eirin A, Bell CC, Crane JA, Tang H (2012). Humanin prevents intra-renal microvascular remodeling and inflammation in hypercholesterolemic ApoE deficient mice. Life Sci.

[25] Sastre C, Rubio-Navarro A, Buendia I, Gomez-Guerrero C, Blanco J, Mas S (2013). Hyperlipidemia-associated renal damage decreases Klotho expression in kidneys from ApoE knockout mice. PLoS One.

[26] Georgescu A, Alexandru N, Andrei E, Dragan E, Cochior D, Dias S (2016). Effects of transplanted circulating endothelial progenitor cells and platelet microparticles in atherosclerosis development. Biol Cell.

[27] Haghighat A, Weiss D, Whalin MK, Cowan DP, Taylor WR (2007). Granulocyte colony stimulating factor and granulocyte macrophage colony-stimulating factor exacerbate atherosclerosis in apolipoprotein E-deficient mice. Circulation.

[28] Chade AR, Zhu X, Lavi R, Krier JD, Pislaru S, Simari RD (2009). Endothelial progenitor cells restore renal function in chronic experimental renovascular disease. Circulation.

[29] Chade AR, Zhu XY, Krier JD, Jordan KL, Textor SC, Grande JP (2010). Endothelial progenitor cells homing and renal repair in experimental renovascular disease. Stem cells (Dayton, Ohio).

[30] Sun YB, Qu X, Li X, Nikolic-Paterson DJ, Li J (2013). Endothelial dysfunction exacerbates renal interstitial fibrosis through enhancing fibroblast Smad3 linker phosphorylation in the mouse obstructed kidney. PLoS One.

[31] Qu X, Jiang M, Sun YB, Jiang X, Fu P, Ren Y (2015). The Smad3/Smad4/CDK9 complex promotes renal fibrosis in mice with unilateral ureteral obstruction. Kidney Int.

[32] Feng M, Tang PM, Huang XR, Sun SF, You YK, Xiao J, Lv LL, Xu AP, Lan HY (2018). TGF-beta mediates renal fibrosis via the Smad3-Erbb4-IR long noncoding RNA Axis. Mol Ther.

[33] Shuai L, Li X, He Q, Dang X, Chen H, Zhou P (2012). Angiogenic effect of endothelial progenitor cells transfected with telomerase reverse transcriptase on peritubular micro vessel in five out of six subtotal nephrectomy rats. Ren Fail.

